# Optimizing Air Scouring Energy for Sustainable Membrane Bioreactor Operation by Characterizing the Combination of Factors Leading to Threshold Limiting Conditions

**DOI:** 10.3390/membranes14030058

**Published:** 2024-02-23

**Authors:** Changyoon Jun, Kimia Aghasadeghi, Glen T. Daigger

**Affiliations:** 1 Department of Civil and Environmental Engineering, University of Michigan, Ann Arbor, MI 48109, USA; gdaigger@umich.edu; 2 Fibracast Ltd., Hannon, ON L0R 1P0, Canada; kimia.aghasadeghi@fibracast.com

**Keywords:** membrane bioreactor, critical flux, threshold flux, air energy saving, critical air flow

## Abstract

Key operating variables to predict the necessary scour air flowrate in full-scale Membrane Bioreactor (MBR) systems are identified, aiming to optimize energy consumption while avoiding the limiting condition (i.e., rapid increasing total resistance). The resulting metric, referred to here as the K value, was derived by balancing hydrodynamic conditions between the particle deposit rate imposed by permeate flux normalized by fouling condition and its removal by shear stress induced from air scouring. The metric includes air scouring flow, permeate flow, Mixed Liquor Suspended Solids (MLSS) concentration, Mixed Liquor (ML) viscosity, membrane packing density, and total resistance. Long-term (year-long) data from two full-scale MBR plants were analyzed. The value of K corresponding to limiting operational operation and referred to as the limiting K value, K_Lim_, is estimated by detecting the occurrence of threshold limiting flux from the data stream and calculating the resulting value for K. Then, using K_Lim_, the minimum required specific air demand per permeate (SAD_p,Crit_) is calculated, indicating a potential reduction of over half the air scouring energy in typical operational conditions. The results from this data driven analysis suggest the feasibility of employing K_Lim_ to predict the adequate scour air flowrate in terms of dynamically varying operational conditions. This approach will lead to the development of energy-efficient algorithms, significantly reducing scour air energy consumption in the full-scale MBR system.

## 1. Introduction

The Membrane Bioreactor (MBR) process provides important benefits, including high effluent quality and stable operation. These benefits have led to the MBR process emerging as a key technology for sustainable water management, particularly for water reuse. MBRs also enable decentralized water management, allowing for more localized and efficient water management strategies [1]. Despite the growing use of MBRs, persistent barriers to its wider use exist, including operational difficulties such as managing fouling and high energy consumption [2]. MBRs require energy to filter Mixed Liquor Suspended Solids (MLSS) to produce permeate by applying Transmembrane Pressure (TMP) across the membrane surface area, often via a pump. This results in material deposition on the membrane surface. Simultaneously, it is imperative to remove depositing materials from the membrane surface to allow stable permeation, and this is accomplished in submerged membranes by generating a crossflow to the membrane surface via air bubble scouring.

The energy required for pumping (i.e., TMP) for permeation under constant flux operation becomes a dependent and uncontrolled variable, varying primarily with the applied flux, the characteristics of the Mixed Liquor (ML), and membrane condition. In contrast, the energy for air scouring in the MBR tank is a controllable variable accounting for approximately one third of the total energy consumption in a full-scale MBR system [3]. The design scour air flowrate is typically determined empirically by the system provider using multiple criteria, such as Specific Aeration Demand per Membrane surface area (SAD_m_, [m^3^/m^2^/h]), or Specific Aeration Demand per Permeate (SAD_p_ [m^3^-air/m^3^-permeate]) [4].

Process designs must consider a variety of limiting conditions, which for the required scour air flowrate can include the peak permeate flowrate, lowest temperature, and the poorest (most fouled) membrane condition allowable. A safety factor (e.g., margin of safety) may also be added to account for uncertainty. Since this combination of factors will occur only infrequently, the required scour air flowrate, and consequently the energy for air scour, will generally be less than the design value. This suggests that constant and rigorous air scouring, irrespective of dynamically varying operational conditions, is unnecessary and will result in unneeded energy consumption. Development of guidelines to determine the appropriate scour air flowrate under specific operating conditions could help reduce unnecessary energy consumption. Excessive air scouring can also adversely impact membrane filtration conditions by increasing colloidal particle accumulation on/in the membranes, promoting severe fouling [5,6,7]. Indeed, it has also been reported that, beyond a certain scour air flowrate, the increase in shear stress effectiveness becomes insignificant [8,9]. Hence, an optimized control strategy for the scour air flowrate can enable the dual objectives of energy savings and stable operation to be achieved.

Determining the optimal air scouring strategy is a non-trivial problem due to the highly complex nature of MBR systems. Temporal and spatially varying operational conditions necessitate different required air scouring levels to prevent solid deposition onto the membrane under particular conditions. The concept of ‘critical flux’, proposed by Field [10], suggests a fundamental concept explaining this complexity. It can be determined empirically, usually by the flux-step method [11], particularly by monitoring the increase in TMP rate over a certain time window [12]. Critical flux phenomenologically represents the initiation of particle deposition where the hydrodynamic balance between deposition and scouring becomes unbalanced, thus allowing solids to accumulate, which in turn is manifested by increasing resistance (or increasing TMP during a given permeation cycle). However, the concept of critical flux, while of theoretical importance, often encounters practical difficulties in its application within the operational conditions of full-scale MBR systems, due to the continuous occurrence of particle deposition and adsorption that persist even in typical operational conditions. A more practical term, ‘Threshold flux’, was suggested by [13], which allows for modest fouling while highlighting a rapid increase in resistance as a threshold point. Here, we focus on the threshold flux concept and apply it to characterize the occurrence of limiting conditions in submerged MBR systems (i.e., differentiate threshold flux to critical flux). Even though the literature has concentrated on the concept of critical flux, the relevant observations are reasonably applicable to threshold flux, as both are part of the same continuum.

Controlled laboratory-scale experiments have evaluated parameters affecting scour air requirements to avoid critical flux, including MLSS, shear stress, and viscosity [14,15,16,17]. These studies have not evaluated long-term performance of full-scale facilities, but it is encouraging that these experimental results indicate the importance of these variables in determining the conditions resulting in critical flux. However, determining critical flux (or threshold flux) and the corresponding specific air demand in a full-scale MBR system poses a system burden and challenges in accounting for dynamically varying operational conditions. Indeed, Monclus et al. suggests that the flux step method tends to conservatively estimate critical flux, often resulting in a lower SAD_m_ compared to critical flux determination using the aeration-step method, even under the same operational conditions [18]. This discrepancy may be primarily due to different underlying fouling conditions, highlighting the difficulties applying measurements made under specific operational conditions to the highly dynamic systems encountered in full-scale applications where conditions are continuously changing.

Here we examine a solution to the challenging task of optimizing air scour energy consumption in a full-scale MBR system. To begin, we consider the threshold boundary in the relationship between the applied shear forces and particle deposition rate under a given operational condition, beyond which rapidly increasing resistance is likely to occur. While this threshold relationship should occur consistently, variability in observations often leads to inconsistent numerical values, such as different SAD_p_. By estimating an underlying limiting boundary, we can determine the critical air scouring flowrate for a particular set of operating conditions while preventing the limiting condition (i.e., threshold limiting flux). Our objective is to develop an approach that can be practically applied in full-scale MBR systems, effectively guiding the determination of SAD_m_ or SAD_p_.

In this paper we develop and define a metric, K, based on the assumptions described above, to characterize the conditions under which the threshold limiting flux will occur and test this concept using data from full-scale MBR plants. An algorithm to detect the occurrence of the threshold limiting flux from available plant operating data is developed. The numerical value of K is estimated under the identified threshold limiting conditions, $\hat{K}$_Lim_, and it is used to distinguish between sets of operating conditions where threshold limiting flux is and is not likely to occur. Lastly, determined values of $\hat{K}$_Lim_ are used to estimate potential energy savings associated with operation at the lower scour air flowrates.

## 2. Materials and Methods

### 2.1. Development of Relationship Characterizing Limiting Condition

As discussed above, here we define the threshold flux as the condition where suspended solids are being deposited on the membrane surface at a significantly higher rate than they are being removed, resulting in a rapidly increasing resistance unless appropriately managed. This contrasts with membrane fouling, which consists of adsorption, pore blocking, sludge deposition (commonly referred to as cake fouling), and inner dense (gel-like) layer formation [19,20], all of which contribute to the total resistance but occur over longer timeframes.

A submerged permeation cycle typically lasts several minutes, which is relatively short compared to the diurnal variations in influent flow (i.e., a few hours). As a result, permeate flux and permeate viscosity do not vary significantly within the timescale of individual permeation cycles. Under these conditions, the total resistance is generally constant in a permeation cycle unless cake fouling rapidly develops. Therefore, TMP varies linearly with imposed flux and fouling condition (i.e., total resistance) following Darcy’s law, as represented by Equation (1):

(1) $$J=\frac{TMP}{\mu_{w}\times R_{T}}$$

where J = Q_P_/A (permeate flow/Area) [m^3^/(m^2^·h)], R_T_ [m^−1^] represents the total resistance to mass transfer of the membrane, and $\mu_{w}$ [Pa·s] is the viscosity of permeate water, corrected to a standard temperature of 20 °C.

The scour air flowrate must be sufficient to remove the solids depositing onto the membrane surface during a permeation cycle as fast as they are being deposited to avoid threshold flux. We characterize the energy applied to the membranes based on the standard definition of the velocity gradient [21], G [s^−1^], as expressed in the following Equation (2):

(2) $$G={\left(\frac{P}{V\times \mu_{abs}}\right)}^{0.5}$$

where P [(kg·m^2^)/s^3^] is the power dissipated in a volume (V [m^3^]) and $\mu_{\mathrm{abs}}$ [Pa·s] is the absolute viscosity of the fluid in the volume of concern. Recognizing that the power dissipated in the volume of the membrane units varies in proportion to the scour air flowrate (Q_A_ [m^3^/h]) [22], this term can replace P in Equation (2). In addition, considering the non-Newtonian rheological characteristics of ML, which are influenced by shear stress [22], $\mu_{\mathrm{abs}}$ is more accurately replaced by apparent viscosity ($\mu_{\mathrm{app}}$).

The energy for air scouring must be sufficient to remove the solids being deposited on the membrane surface, solid flux, as follows:

(3) $$Solid\;Flux=\frac{MLSS\times {\;Q}_{P}}{A}\;$$

At the limiting condition, the velocity gradient applied must be greater than the applied solids flux, as follows:

(4) $${\kappa_{1}\left(\frac{Q_{A}}{V\times \mu_{app}}\right)}^{0.5}\geq \;\kappa_{2}\frac{MLSS\times Q_{P}}{A}$$

where $\kappa_{1}$ and $\kappa_{2}$ are constants of proportionality.

Equation (4) is an earlier version of the relationship [23] and it has been extended to incorporate the condition of the membrane as characterized by the total resistance term, R_T_ (i.e., membrane fouling condition, and inverse of permeability). We tested numerous relationships to determine the best representation of this proportionately using the data sets used in this research and described below. A summary of this analysis is presented in the Supplementary Materials, within the section titled ‘Different Version of K Value Equations’ and ‘Different Versions of K value Equations’. We found that including membrane resistance was necessary to reflect the condition of the membranes. The following equation was adopted based on this analysis, representing the relationship between scour air flowrate and applied solids flux:

(5) $$\frac{Q_{A}}{V\times \mu_{app}}\geq \;K_{Lim}\times MLSS\times \frac{Q_{P}}{A}\times R_{T}$$

where K_Lim_ = $\kappa_{2}/\kappa_{1}$ [(m·s)/kg] is a constant representing the critical relationship between the shear intensity applied to remove deposited solids and the rate of solids deposition. In other words, K_Lim_ represents a threshold relating the balance of air scouring with solids deposition below which the limiting condition (threshold flux) occurs.

Rearranging Equation (5) to define K_Lim_ gives the following:

(6) $$K_{Lim}\leq \alpha_{s}\times Q_{A}\times \frac{1}{Q_{P}\times \mu_{app}\times MLSS\times R_{T}}$$

where $\alpha_{s}$ [m^−1^] represents the membrane packing density (A/V), and $\mu_{w}$ [Pa·s] is the temperature corrected viscosity of water under standard conditions. The right-hand side of Equation (6) can be calculated for any combination of operating conditions and, for a resulting numerical value exceeding the value of K_Lim_ for a particular application, threshold limiting flux will be avoided. Equation (6) can also be used to estimate $\hat{K}$_Lim_ if the combination of operating conditions resulting in threshold limiting flux is known.

The MLSS concentration of the membrane influent for MBR applications is high enough to affect the viscosity of the fluid in the membrane bundle. Several correlations are available from the literature relating the viscosity of the activated sludge ML to the MLSS concentration and other variables. A representative example is the Ostwald equation [22], as Equation (7):

(7) $$\mu_{app}=fn(MLSS,\;G)=\exp\left(1.71\times {MLSS}^{0.45}\right)\times G^{\left(-0.068\times {MLSS}^{0.81}\right)}$$

Replacing R_T_ by (TMP·A)/(Q_P_ ·$\mu_{w}$) and setting the left- and right-hand sides of Equation (6) as equal, the critical scour air (Q_A,Crit_) can be determined. This result is expressed as the ratio of the critical air scour flow to the permeate flow (i.e., Critical Specific Air Demand per Permeate, SAD_p,Crit_ = Q_A,Crit_/Q_P_) below:

(8) $$\frac{Q_{A,Crit}}{Q_{P}}\;=\frac{K_{Lim}}{\alpha_{s}}\times MLSS\times \mu_{app}\times \left(\frac{TMP\times A}{Q_{P}\times \mu_{w}}\right)$$

Equation (8) applies to a specific membrane configuration and process application. For example, the uniformity with which the scour air is applied will affect the numerical value of K_Lim_, as deposited solids must be efficiently removed throughout the membrane bundle [24]. Referring specifically to Equation (8), SAD_p,Crit_ is proportional to the site-specific constant term (K_Lim_/$\alpha_{s}$). Higher fouling conditions increase the hydrodynamic barrier, thus raising the local flux leading to a higher deposition rate than the actual imposed flux [25]. Indeed, the total resistance term in Equation (5) normalizes the measured permeate flux to the local flux. Consequently, a higher scour air flowrate is required under higher fouling conditions [26], indicated in this relationship by the proportional total resistance term. The characteristics of the ML rheology term ($\mathrm{MLSS}\cdot \mu_{\mathrm{app}})$ for the ML being filtered also affects the scouring intensity needed for their removal.

This development suggests that SAD_p,Crit_, as expressed by Equation (8), can be viewed as a practical operating parameter to minimize the air scour flow rate. This parameter enables estimation of the minimum required air scouring flow rate for a given permeate flow rate, which can be computed from online measurements and site-specific parameters. The site-specific K_Lim_ value, coupled with Equation (8), allows determination of SAD_p,Crit_ as a function of a dynamically varying combination of factors based on site specific constants, ML rheology, and the fouling condition of a given system. Knowledge of K_Lim_ for a particular installation allows estimation of potential energy savings associated with operating at the lower boundary of air scouring flow rates, rather than maintaining a fixed air scouring set point.

### 2.2. Full-Scale MBR Systems Evaluated

The data used in this study is from two full-scale MBR systems treated with municipal wastewater, hereafter referred to as plant A and plant B. The data incorporate operational periods, from 1 July 2022 to 15 August 2023 for both plant A and plant B. Both plants have MBR systems equipped with FibrePlate™ membrane technology by Fibracast. These membranes integrate hollow-fiber and flat-sheet technologies. A FibrePlate™ module consists of PVDF membrane sheets comprised of permeation channels aligned horizontally. The modules have vertical headers and are mounted horizontally into the cassettes, which allows a vertical ML flow path incorporating influent and Return Activated Sludge (RAS) flow (Q_R_ [m^3^/h]) that is free from header obstructions [27]. The FibrePlate™ membrane configuration also results in a higher packing density, which can reduce scour air flowrates as suggested by Equation (8) above.

Information on the MBR configuration for the two plants is summarized in Table 1. The operational protocols are programmed to have production cycles consisting of a period of permeation followed by a period of relaxation and/or back pulse. The permeate pumps stop during the relaxation mode, but the scour air and RAS flow continue. The direction of flow in the membrane is reversed during the back pulse mode from outside-in to inside-out, using permeate water for back pulsing. Table 2 provides the production cycle information for each plant. The membrane systems require weekly maintenance cleanings and recovery cleaning every 6–12 months. Maintenance cleaning involves ten 1-min back pulses using water mixed with chemicals (300 mg/L hypo, 2000 mg/L citric acid). Recovery cleaning involves an 8–12 h soak in hypochlorite (1000 mg/L) followed by an 8–12 h soak in citric acid (2000 mg/L). While both plants have similar maintenance protocols, their operating conditions vary. For instance, maintenance cleanings and recovery cleanings differ based on operating requirements and the practices of the operators at each specific facility. The air flow set-point is continuously maintained at 0.110 m^3^/m^2^/h, and 0.165 m^3^/m^2^/h above a permeate flux of 12.3 LMH for Plant A, while Plant B has a single air flow set-point of 0.147 m^3^/m^2^/h.

### 2.3. Data Analysis

Online measured data was collected, including TMP [kPa], permeate flow rate [m^3^/h], scour air flowrate [m^3^/h], RAS flowrate [m^3^/h], and temperature [°C]. Laboratory data, measured periodically on samples from the bioreactor prior to the MBR tank, were also available, including MLSS concentration, RAS flow to Permeate flow ratio (RAS Q [m^3^-RAS/m^3^-Permeate]), time to filter (TFF) [s], and solid retention time (SRT) [day]. The laboratory data was collected approximately once every two weeks. MLSS concentration values between the measured values were filled based on the previous measured value first, and then the next value if the ahead value was not available. This is reasonable as the characteristics of ML vary gradually. Table 3 summarizes the operational conditions in terms of ML characteristics for the two plants.

### 2.4. Development of the Algorithm for Cycle Extraction and Detection of Limiting Condition

The collected dataset contains continuously measured values, necessitating preprocessing. An algorithm, referred to as the “Extraction Algorithm”, was developed for this purpose. It was designed to extract each cycle from the sequential data stream and pre-filter it by validating whether it reflected actual operational conditions before storing each cycle separately. The algorithm consists of three-phase data processing; (I) detect starting point and end point of each permeation cycle; (II) extract cycle and polishing noises; and (III) post-polishing the extracted cycles, in which non-representative, out of operation, and outliers are filtered and excluded for further analysis. The algorithm then determined whether the limiting condition occurred during each permeation cycle by computing the fouling rate (ΔTMP/Δt) over that permeation cycle as suggested method by Le Clech et al. [11].

If all fouling rates in the last one and a half minutes were higher than a criterion, 0.017 kPa/s, the cycle was classified as ‘Limiting Condition’ (i.e., threshold limiting flux). If all were below this criterion, it was labeled as ‘Sub-Limiting Condition’. If the extracted cycles did not belong to either class, they were classified as ‘Undefined’. This classification helped reduce false-positive cycles. The criterion of 0.017 kPa/s was proposed by [28], and significant effort was invested to empirically evaluate this and the other criteria used. Algorithm validity was verified by checking individual points to confirm whether limiting or sub-limiting conditions were correctly labeled. A limited number of false positives and false negatives could occur with this algorithm due to stochastically fluctuating operational conditions and latent noise; however, it proved reasonably effective at capturing the limiting condition. The sample of extracted cycles identified as threshold limiting flux by the Extraction Algorithm, along with variables (TMP, Flux, and permeability) is presented in the ‘Variation of Featured Variables in Limiting Conditions’ section of the Supplementary Materials.

K values were computed based on the right-hand side of Equation (6) and labeled for both limiting and sub-limiting conditions, referred to here as K_Lim_ and K_SubLim_, respectively. The value of $\mu_{\mathrm{app}}$ was calculated using Equation (7). Values for G are required for this equation. The G value for air flow was calculated based on Delgado et al. [22] as described in Equation (9):

(9) $$G_{Air}={\left(\frac{1000}{\exp\left(1.71\times {MLSS}^{0.45}\right)}\cdot \frac{Q_{A}\times \gamma_{l}\times h}{60\times V}\right)}^{1/\left(-0.068\times {MLSS}^{0.81}+2\right)}$$

where $\gamma_{l}$ is liquid specific weight [N/m^3^], h is liquid depth over the scour air diffusers [m], and V is column volume [m^3^]. The contribution of ML flow must be added to that for scour air as the ML also flows through the membrane bundles.

The G value for ML flow was computed using the ML flow rate. The flow rate per module was divided by the cross-sectional area of a module to compute the ML flow velocity, and subsequently the energy imparted to the fluid within the membrane module was determined, as described in Equation (10):

(10) $$G_{ML}=\left({\frac{1000\times P_{ML}}{V_{col}\times \mu_{app}}}^{0.5}\right)$$

where $P_{\mathrm{ML}}$ is hydraulic power of the up-lifting ML flow (i.e., RAS flow and influent flow).

### 2.5. Validation of K Value as a Metric to Estimate Limiting Condition

K_Lim_ and K_SubLim_ were further analyzed on a statistical basis to evaluate their effectiveness at predicting limiting conditions. The assumption is that the classified K values come from different operational conditions. In other words, K_Lim_ is more likely to be observed when the operational condition is limiting, whereas K_SubLim_ is unaffected by operating conditions. Numerical values of K_Lim_ should be systematically lower than those for K_SubLim_ as operation under limiting conditions corresponds with operating at the lowest feasible scour air flowrate, while avoidance of limiting conditions (the occurrence of sub-limiting conditions) is achieved by operating at higher scour air flowrates (see Equation (6)). Consequently, K_Lim_ should be capable of differentiating between limiting and non-limiting conditions. To evaluate this assumption, kernel density distributions for K_Lim_ and K_SubLim_ were compared, and further statistical analyses were carried out to determine whether the two classes originate from different populations. Then, ${\hat{K}}_{\mathrm{Lim}}$ was estimated and statistically validated using the Coefficient of Variation (CV) by comparing the standard deviation of K_Lim_ to the mean of K_Lim._

### 2.6. Estimation of Reducible Air Scouring Energy with K_Lim_

The controllable variable for the membrane system in an operating MBR is the scour air flowrate. Given a site-specific value of ${\hat{K}}_{\mathrm{Lim}}$, Equation (8) can be used to calculate the critical scour air permeate flowrate for a particular set of operating conditions for each permeation cycle, SAD_p,Crit_. Comparing this to historical SAD_p_ data provides a general guideline for the minimum scour air flowrate needed. Additionally, to monitor the trends of SAD_p_ variation, a moving average of observed SAD_p_ using a 14-days window is calculated as follows:

(11) $$\;{MA(SAD)}_{p}=\frac{1}{N}\times \sum_{i=1}^{N}{{SAD}_{p,i}}_{\;}$$

where MA(SAD)_p_ is the moving average of SAD_p_ for a given day, N is number of samples in the window size, and SAD_p,*i*_ is *i*-th observed SAD_p_ within this window.

## 3. Results

### 3.1. Summary of Plant Operational Conditions

**Figure 1:**
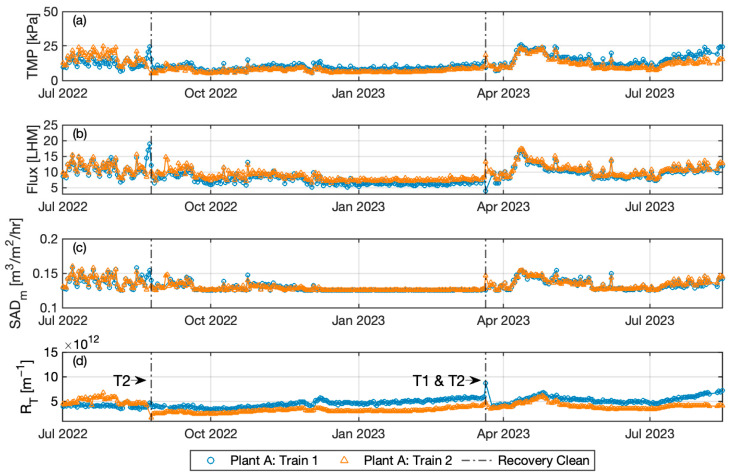
Time series variation of (**a**) daily averaged TMP, (**b**) permeate flux, (**c**) specific air, and (**d**) total resistant in Plant A. Vertical dash-dot lines represent the date of recovery clean.

**Figure 2:**
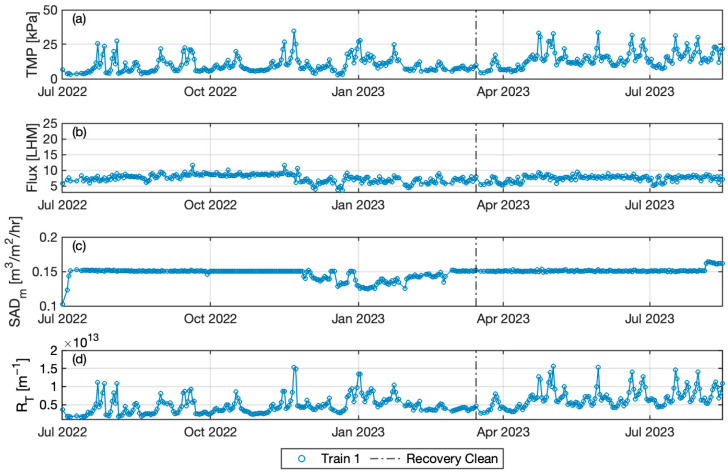
Time series variation of (**a**) daily averaged TMP, (**b**) permeate flux, (**c**) specific air, and (**d**) total resistant in Plant B. Vertical dash-dot line represents the date of the recovery clean.

Figure 1 and Figure 2 display the time-series variation of the daily average TMP, permeate flux, specific air flux, and total resistance for Plant A Trains 1 and 2, and Plant B, respectively. Seasonal variation in the permeate flux is observed for Plant A, with values approaching the design value, while permeate flux is lower for Plant B. The TMP for Plant A Train 2 in the initial period is higher than Train 1, corresponding to the higher total resistance in this early period. After the recovery clean at the end of August 2022, Train 2 shows generally lower TMP throughout the remaining observation period. Permeate flux is higher in Train 2, especially after August 2022. Permeate flux displays a valley-like swing profile due to a lower influent flowrate in the middle of the period, a systemic variation caused by the dry season. Permeate flux is very low from December 2022 to March 2023, leading to stable operating conditions. This corresponds to a relatively consistent variation in daily averaged TMP during this period. The influent flow increases from March onwards due to melting snow. TMP increases accordingly in both trains but is higher in Train 1, even with a slightly lower permeate than that in Train 2, likely due to different degrees of fouling. Accordingly, total resistance is higher in Train 1 than Train 2. Recovery cleans were performed twice in Train 2 (August 2022 and March 2023), while only once in Train 1 in March 2023. All these recovery cleanings effectively reduced the total resistance. Airflow variation is similar in both trains, as the scour air flowrate in Plant A has two set points depending on permeate flux. Thus, during the dry season, the scour air flowrate is maintained at a lower value.

The TMP periodically and rapidly increased in Plant B, even with a relatively consistent and lower permeate flux and higher scour air flux in comparison to Plant A. As shown in Table 4, the mean permeate flux for Plant B was 7.4 LMH, lower than both trains of Plant A at 8.8 LMH and 9.6 LMH, respectively, while the daily averaged SAD_m_ was higher in Plant B at 0.15 m^3^/m^2^/h than at 0.13 m^3^/m^2^/h in both Trains 1 and 2 of Plant A. The daily averaged SAD_p_ was 20.6 for Plant B but 15.8 and 14.2 for both trains of Plant A, respectively. Temporal spikes occurred once TMP profiles started to increase, which were mitigated after chemical cleaning events (chemical cleaning is not shown here), suggesting Extracellular Polymeric Substances (EPS)-related fouling. The increasing resistance profile compares well with the resistance curve caused by EPS accumulation reported by Nagaoka et al. [29]. As discussed below, these spikes generally coincided with the occurrence of limiting condition and returned to near previous values after the maintenance clean, suggesting that rapid fouling was generally reversible. The TMP level following chemical cleaning gradually increased, however, indicating that some irreversible fouling occurred. This is a well-known effect of limiting conditions and high TMP, which can accelerate formation of intact fouling layers [30].

Despite operating under apparently more favorable conditions than Plant A, Plant B experienced threshold limiting flux more frequently. Table 5 provides a summary of observed threshold limiting flux occurrences, which align with these findings as the number of the observed threshold limiting flux for Plant B is higher than Plant A. Several potential factors may contribute to the more frequent occurrence of threshold limiting flux for Plant B. Plant B carries out chemical cleaning every 4–6 days, while Plant A does so every 3 days. Plant A also has a relaxation and back pulse cycle while Plant B only has relaxation. This difference in cleaning protocols could result in varying quantities of accumulated EPS on the membrane in Plant B. Accumulated EPS can cause a denser and less porous cake layer, greatly increasing resistance [31], and can induce a threshold limiting flux under comparatively lower permeate flux conditions [32]. Secondly, there may be inherent differences in the influent wastewater characteristics and bioreactor configuration, leading to increased Soluble Microbial Product (SMP) concentrations and EPS production at Plant B than that at Plant A. Thirdly, a systematic difference in the effectiveness of air scouring due to different system configurations may exist between the two plants, which could result in lower air scouring effectiveness at Plant B. Finally, a higher air to permeate flow rate could increase the amount of fine material, such as colloidal material, and lead to more rapid fouling. Though these observations are not the central focus of this study, they highlight the difference in fundamental behavior for these two facilities.

### 3.2. Time Series Variation of K Value

**Figure 3:**
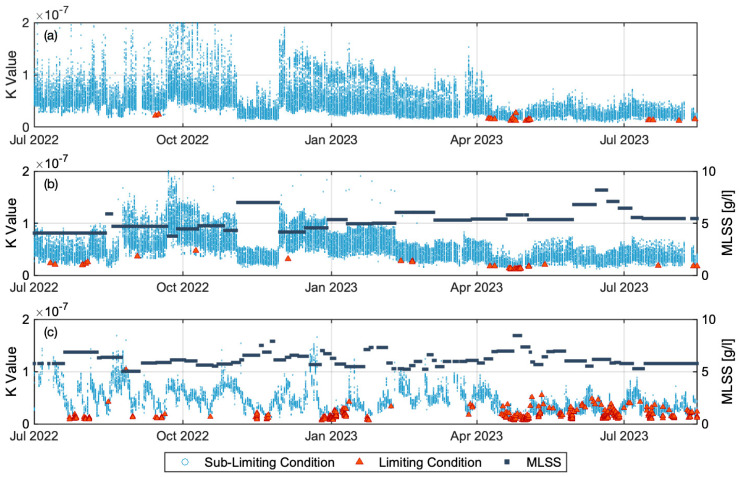
Time series variation of K values in limiting condition (Red triangle), sub-limiting condition (Blue dot), and MLSS concentration (Black square): (**a**) Plant A Train 1, (**b**) Plant A Train 2, and (**c**) Plant B Train 1.

Figure 3 illustrates the time series variation of the K values computed according to Equation (6). K_Lim_ occurs consistently within the lowest range of calculated K values while values of K_SubLim_ are consistently higher, as expected. A higher K value suggests that the applied scour air flowrate may be higher than needed, particularly due to lower permeate flux compared to the design flux. The K value for Plant A decreases during the later period, which is attributed to increased permeate flux due to rising influent flow from melting snow. A lower K value indicates an operation closer to the threshold limiting flux, which occurs for a combination of factors as defined by those included in Equation (6). Consequently, the probability of observing threshold limiting flux is increased. In contrast, higher irreversible fouling existed in the early period of Train 2 of Plant A than Train 1, which led to Train 2 being more prone to the occurrence of threshold limiting flux under similar mean permeate flux. Recovery cleans of both trains at the end of March 2023 would have removed accumulated foulants, such as pore blocking and gel layer [33] and restored similar conditions to the membranes in both trains. The sudden increase in permeate flux to 23 LMH in April and May resulted in the occurrence of threshold limiting flux in both Trains of Plant A.

K value profiles varied differently for Plant B than for Plant A, characterized by periodically repeating decreases in K that reached K_Lim_, representing the occurrence of threshold limiting flux. These events were associated with abrupt TMP increases, indicating that the K value profiles were associated with fouling conditions as characterized by increasing resistance. This is unlike Plant A, where the occurrence of threshold limiting flux occurs because of increased flux. Plant B started operation from June 2022 and initially demonstrated similar or higher K values compared to Plant A. The computed value of K decreased rapidly, but was associated with a rapid increase in TMP and resistance (Figure 3c).

### 3.3. Distinguishing Limiting versus Sub-Limiting Operating Conditions Using K_Lim_

**Figure 4:**
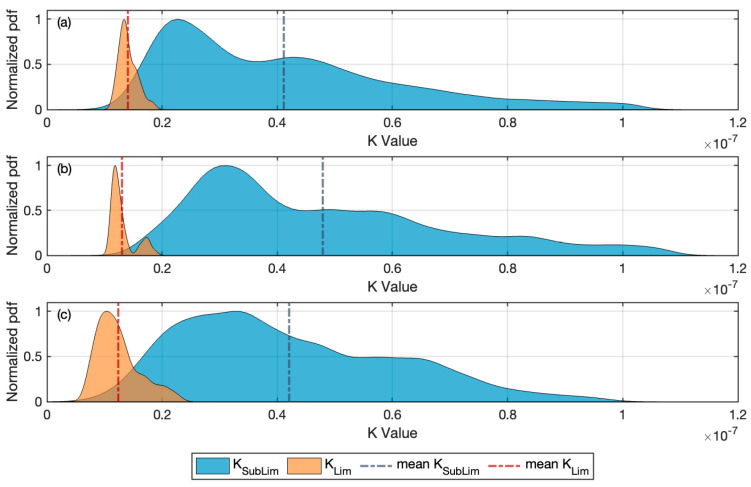
Comparison of normalized kernel density distribution for K Values: (**a**) Plant A Train 1, (**b**) Plant A Train 2, and (**c**) Plant B Train 1. Vertical red dotted line and blue dotted line represent the arithmetic average for K_Lim_ and K_SubLim,_ respectively.

Figure 4 presents the distributions of K_Lim_ and K_SubLim_ values derived using Kernel Density Estimation. The results indicate a narrow range of measured values of K_Lim_, with a distinctly smaller range than the reported values of K_SubLim_, and minimal overlap which supports the capability of calculated values of K to discriminate limiting from sub-limiting operating conditions. K_Lim_ is a variable representing a defined and specific set of operating conditions (those resulting in threshold limiting flux), while K_SubLim_ simply represents a combination of operating conditions imposed on the system. If we consider these variables to be compared and conduct a two-sample Kolmogorov–Smirnov (K-S) test reflecting the non-normal nature of the variability in each variable, we find that the resulting *p*-value close to zero indicates a statistically significant difference.

Table 6 summarizes the statistics for K_Lim_ and K_SubLim_. Reflecting on the mean values of K_Lim_ for each dataset, they are of similar magnitude but with the value for Plant B being somewhat smaller than the values for the two trains in Plant A. Modest differences can be expected due to differences in ML characteristics (between Plant A and B), fouling conditions, and potential differences in the module configuration. The low CV value for ${\hat{K}}_{\mathrm{Lim}}$ for each system suggests these estimates provide a reasonably precise estimate of K_Lim_.

### 3.4. Estimation of Limiting Scour Air Energy and Its Implications

**Figure 5:**
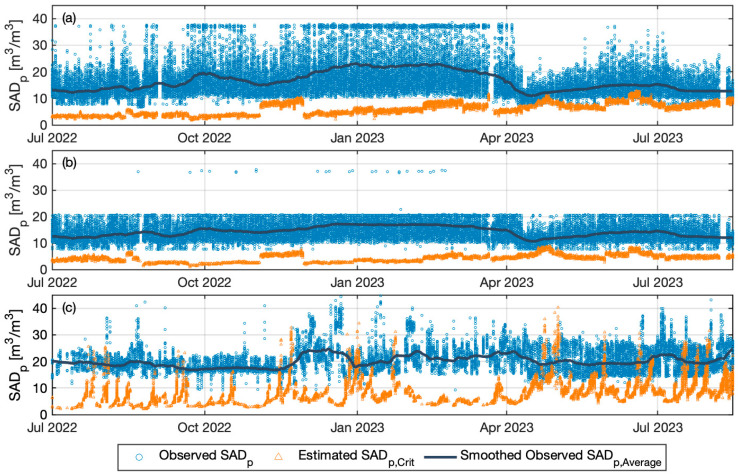
Comparison of actual SAD_p_ (Circles) with predicted SAD_p,Crit_ (Triangles), and smoothed SAD_p,Average_ (Line): (**a**) Plant A Train 1, (**b**) Plant A Train 2, and (**c**) Plant B Train 1.

The limiting scour air flowrate required to avoid threshold limiting flux was calculated by incorporating ${\hat{K}}_{\mathrm{Lim}}$ into Equation (8). Figure 5 compares the estimated SAD_p,Crit_ to the actual operating values. The results suggest that the mean of the estimated SAD_p,Crit_ is 40 (±20), 31 (±14), and 34 (±15) percent of the SAD_p_ for Plant A Train 1, Train 2, and Plant B, respectively. It may be prudent to determine SAD_p_ by adding an appropriate safety factor based, for example, on operating experience. The results indicate, however, the potential for significant energy savings on an on-going basis.

Comparing the three trains, the higher resistance results in higher SAD_p,Crit_. For instance, from Plant A, SAD_p,Crit_ is estimated higher at Train 2 initially, but after recovery cleaning of Train 2 in August, 2022, it was consistently lower than Train 1 until the next recovery cleaning in March, 2023. Higher permeate flow in the period after April 2023 reduced the actual SAD_p_ and correspondingly increased the estimated SAD_p,Crit_, leading to the SAD_p,Crit_ approach the value of the smoothed observed SAD_p,Average_. This, in turn, suggests that the applied air scouring was limited during these periods, which aligns with the results of the frequency of limiting conditions detected, as shown in Table 5. The estimated SAD_p,Crit_ fluctuates much more widely for Plant B, attributable to the periodically repeated fouling. Due to the rapid increase in resistance, estimated SAD_p,Crit_ exceed the SAD_p,Average_. Cho and Fane [34] have experimentally suggested that a cleaning event followed by rapid fouling, characterized by an increase in TMP, may be due to conditions related to EPS. Indeed, under more highly fouling membrane conditions, it is suggested to prioritize cleaning events over increasing the airflow rate as the estimated required scour air flowrate can increase exponentially to prevent the threshold limiting flux [6,35]. Significantly, in Plant B a larger gap is observed between the estimated SAD_p,Crit_ to actual SAD_p_ during periods without fouling issues. This suggests that excessive air scouring may have been practiced during those periods.

## 4. Discussion

The results presented here indicate that significant energy savings can be achieved by adjusting the MBR scour airflow rate in response to actual operating conditions. Required air flow requirements were found to be as little as 31 to 40 percent of the actual quantity of air used for the facilities evaluated in this study. This occurred at these facilities even though adjustments in specific airflow were made. A full-scale evaluation by Monclús et al. [36] demonstrated that a 20% reduction in scouring air does not impact permeability when compared to a parallel train with full scour air flowrate. Miyoshi et al. [37] found in a pilot scale MBR that a 20% reduction in permeate flux led to a 50% decrease in the critical scour air flowrate, supporting the possibility of implementing air scour adjustment within proper ranges. These results suggest that fouling and subsequent permeability reduction are independent of air reduction, as long as a sufficient scour air flowrate is applied. These results further support the approach of minimizing scour air usage, whenever possible, for full-scale facilities. Implementing such reductions would reduce MBR energy costs significantly. What is needed is a suitable metric to indicate the scour airflow required.

In this research we further develop a metric, ${\hat{K}}_{\mathrm{Lim}}$, which can be used to determine the MBR scour airflow required to avoid threshold limiting flux based on current operating conditions. It allows determination of SAD_p,Crit_ based on the current sludge rheology and condition of the membranes. It clearly distinguishes between limiting and sub-limiting operating conditions and can be used to estimate the reduced scour airflow possible for a given operating condition. Prediction of the threshold limiting scour air flowrate can then form the basis for subsequent development of an algorithm to minimize scour air energy when possible.

It is significant that the ${\hat{K}}_{\mathrm{Lim}}$ metric was found to be applicable to both full-scale plants evaluated in this research as the mechanisms by which threshold limiting flux occurred for these two plants were different. Threshold limiting conditions occurred for Plant A due to high permeate flux while for Plant B they occurred due to fouling. The fact that the ${\hat{K}}_{\mathrm{Lim}}$ metric was found to function for both plants, and in fact that the numerical values of ${\hat{K}}_{\mathrm{Lim}}$ were similar for both plants, suggests that use of this metric may have reasonably broad applicability. Further insight into the different responses for the two plants can be obtained by a more detailed analysis of the relationship between TMP and flux for individual permeation cycles.

**Figure 6:**
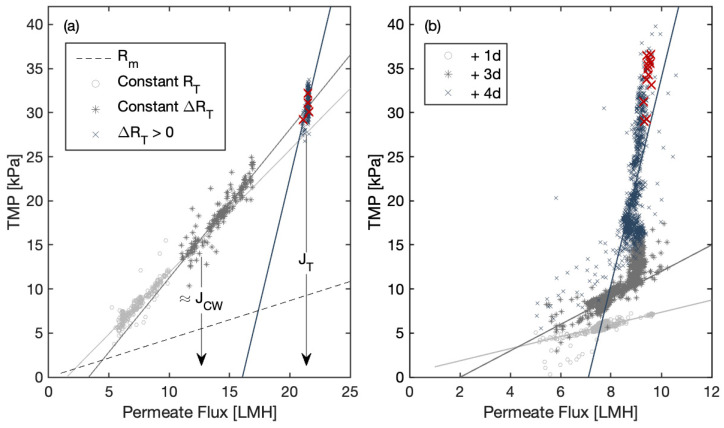
Comparison of specific flux concepts with data collected from the full-scale MBR system: (**a**) data extracted for a day from Plant A, and (**b**) data extracted for four consecutive days from Plant B. The red color marks are detected limiting conditions in both cases. Graphical methodology adapted from [13]. J_CW_ is the weak critical flux, and J_T_ is the threshold flux.

Example plots of TMP versus permeate flux for individual permeate cycles are provided in Figure 6a,b for Plant A and Plant B, respectively. The slope of the line represents R_T_, while the inverse of the slope is the permeability. R_T_ remains relatively stable (ΔR_T_ ≈ 0) under lower flux conditions (below 12 LMH) for Plant A (Figure 6a). Actual operational data are used in these figures, so the first line already contains some fouling, as suggested by the fact that its intercept on the y-axis is not through the origin, but the linear pattern is as expected. R_T_ starts to increase linearly at higher flux, and the intersection between the two resulting lines suggests a weak critical flux was found at 12 LMH as proposed [13]. ΔR_T_ increases non-linearly when the flux exceeds 23 LMH, corresponding to the occurrence of the threshold flux, as highlighted by the red color. In contrast, Plant B (Figure 6b) displays fouling development over four days post a chemical cleaning. TMP began to rise rapidly around 9 LMH on the third day after chemical cleaning, even though the system had been operating under sub-critical conditions on the first day and weak critical flux up to that point. The threshold flux was observed on the fourth day, showing an rapid increase in TMP which deviated substantially from the robust regression line representing the operation on the previous days. Notably, threshold limiting fluxes were observed in both cases once the TMP reached around 30 kPa, suggesting that a threshold pressure triggers the limiting condition.

The example responses presented in Figure 6 further reinforce those different mechanisms resulting in threshold limiting flux development in Plants A and B. The threshold limiting condition occurred in Plant A due to high flux, while the limiting conditions occurred in Plant B at much lower flux but was associated with rapidly increasing TMP for the given flux (i.e., increasing resistance) and varied over time. These differences in response can be interpreted through the Resistance in Series (RIS) model as investigated by others [30,34]. Plant A was predominantly influenced by cake resistance (R_C_), a result of high flux over a relatively short period. In contrast, Plant B experienced internal fouling, such as pore blockage and the formation of denser inner layers, largely attributed to the accumulation of EPS over a relatively long-term period, leading to a progressive increase in local flux. Local flux is essentially a cumulative measure of forces acting on particles near the membrane surface, representing the net convective force [15]. When the local flux surpasses a critical threshold in the presence of a specified counterforce, threshold limiting flux is likely to ensue [25]. This leads to a rapid increase in resistance, with cake resistance becoming the primary contributor to this increase.

These results also indicate the importance of including a term for membrane condition, R_T_ in this case, to account for the extent of fouling of the membranes. As foulants accumulate, required convective forces increase due to thickening concentration polarization, leading to an increase in fouling [38,39], indicating that air scouring should be proportionally increased. In the limiting condition, the required scour air flowrate might increase exponentially because the rapidly increasing convective force leads to higher viscosity, which accelerates further the accumulation of biopolymers and small particles on the membrane surface [26]. Yu et al. [15] theoretically described that higher flux could draw more and denser particles onto the membrane surface, which requires even higher shear velocity to counteract the resulting depositing tendency. The results also suggest that the scour air control algorithm can be effectively applied up until significant fouling occurs, at which point chemical cleaning will need to be prioritized [35].

The MLSS concentration range for the datasets considered here is relatively narrow, ranging from 4 to 6 g/L for Plant A and 5 to 7 g/L for Plant B when threshold limiting flux occurred. This range is well within that typically used for full-scale MBR plants and within the range of 4 to 10 g/L reported to result in limited effects on viscosity and thus permeability [16,26,40,41]. Exponentially increasing viscosity and resistance is often observed for MLSS concentrations exceeding 10 g/L [26]. The use of an empirical equation relating viscosity to MLSS concentration and G value from the literature [22] based on laboratory data is also a constraint. Further characterizing the impacts of MLSS concentration would only become important in applications with widely varying MLSS.

The membranes used in both plants studied in this research utilize two-phase flow incorporating ML flow and scour air flow, compared to other systems using only scour air flow. The direction of ML flow through the membrane bundles may offer some additional advantages relative to the occurrence of the threshold limiting flux. Hydraulic flow through the membrane bundles is controllable, potentially providing a more uniform distribution of shear stress imposed by air scour energy and MLSS concentrations throughout the membrane bundle. ML flow may affect scour air bubble interactions with the membrane surface, which differ by flow type and membrane movement [42]. ML flow is directed upward through the membrane modules, which adds additional shear stress and helps mitigate sludge deposition [20]. It is reported that the development of critical flux varies spatially from the inlet to the outlet of the membrane bundle. With ML flow from the bottom of the cassette, varying TMP conditions from the bottom to the top of the membrane bundle may be reduced, leading to more uniformity [43]. Effects such as these further demonstrate the need for site-specific values of ${\hat{K}}_{\mathrm{Lim}}$, irrespective of the membrane type.

The estimated SAD_p,Crit_ in Figure 5 is computed using Equation (8), which is a linear function of the variables included on the right-hand side. The relationship between the two might not be strictly linear; however, SAD_p,Crit_ might increase at higher values of variable combinations due to factors such as compaction of the fouling layer or reduced effectiveness of shear stress induced by air scouring due to increased viscosity adjacent to the membrane side [39,44]. Conversely, there could be a minimal threshold for SAD_p,Crit_ below which fouling is more likely to occur at lower values of these combinations, as observed experimentally [37,45,46]. Despite these extremes, the typical operational range is likely to generally maintain a linear relationship, supported by results indicating a linear relationship between shear stress and critical flux under different operational conditions [28]. Further data from the actual system is needed to investigate the potential for these effects. Nevertheless, given the main objective to estimate the reducible air scouring flowrate over a normal range of operational conditions, linear estimation is an appropriate starting point for further investigation.

## 5. Conclusions

Data from two full-scale MBR plants were evaluated to identify the combinations of factors leading to threshold limiting flux and resulting in a rapid increase in resistance. The results of this research demonstrate that:

- A factor, referred to here as K_Lim_, representing the minimum scouring air flow rate to net convective force, defines the important parameters and their inter-relationship leading to the occurrence of threshold limiting flux. In addition to the permeate flow, these factors include MLSS concentration, mixed liquor viscosity, membrane packing density, and current operating resistance (or permeability).
- Calculation of the value of K for a particular set of operating conditions and comparison to the site-specific value of ${\hat{K}}_{\mathrm{Lim}}$ can be used to determine whether threshold limiting flux is likely to occur, leading to rapid TMP increase.
- ${\hat{K}}_{\mathrm{Lim}}$ for a particular application might depend, among other factors, on the characteristics of the ML being processed in the system.
- Operation at scour air flowrates based on the limiting value, potentially incorporating a safety factor, can lead to significant membrane operating energy cost savings.

Further work with other full-scale MBRs is needed to further evaluate these results and more fully define the exact nature of the relationship to calculate K value.

## Figures and Tables

**Table 1:** Information on the MBR system analyzed.

Plant ID	Number of Trains	Number of Cassettes per Trains	Total Surface Area per Train [m^2^]
Plant A	2	6	11,710
Plant B	1	3	8480

**Table 2:** Production cycle and cleaning information for the MBR systems.

Plant ID	Permeation Duration [min]	Relax/ Back-Pulse	Relax/Back-Pulse Duration [s]	Maintenance Clean	Recovery Clean
Plant A	5	Relax/ back pulse after 4 cycles	45	One or two per week (citric) One per week (hypochlorite)	August 2022 on Train 2 March 2023 on Train 1 and 2
Plant B	5	Relax/ No back pulse	45	One per week (hypochlorite)	March 2023

**Table 3:** Operational conditions of ML characteristics for the MBR systems.

Plant ID	Bioreactor Process	Temperature [°C]	MLSS [g/L]	RAS Q [m^3^/m^3^]	TTF [s]	SRT [Day]
Plant A	Anoxic/Aerobic *	12–18	3.8–7.0	5	22–61	15–25
Plant B	Aerobic	20–28	5.0–8.5	6	ND	15

* Plant A has A/O process with ferric chloride addition to remove phosphorus; ND indicates that no data are available.

**Table 4:** Summary of statistics for permeation cycles for arithmetic average and one standard deviation.

Plant ID	TMP [kPa]	Flux [LMH]	SAD_m_ [m^3^/m^2^/h]	SAD_p_ [m^3^/m^3^]	R_T_ [10^12^ m^−1^]
Plant A T1	12.0 (±4.5)	8.8 (±2.4)	0.13 (±0.01)	15.8 (±3.4)	4.8 (±0.8)
Plant A T2	10.5 (±4.7)	9.6 (±2.2)	0.13 (±0.01)	14.2 (±2.2)	3.7 (±0.9)
Plant B T1	11.6 (±6.3)	7.4 (±1.2)	0.15 (±0.01)	20.6 (±3.6)	5.5 (±2.7)

**Table 5:** Occurrence of sub-limiting, limiting, and undefined extracted and labelled permeation cycles for Plants A and B.

Category	Plant A T1	Plant A T2	Plant B T1
Sub-Limiting	56,961	63,380	32,086
Limiting	28	60	742
Q3 2022	2	10	63
Q4 2022	-	2	100
Q1 2023	-	3	180
Q2 2023	21	42	317
Q3 2023	5	3	82
Undefined	1123	977	5080

**Table 6:** Summary statistics of ${\hat{K}}_{\mathrm{Lim}}$ and K_SubLim_ for arithmetic average and one standard deviation.

Plant ID	${\hat{K}}_{\mathrm{Lim}}$ 10^−8^ [m·s/kg]	CV_Lim_ [Dimensionless]	K_SubLim_ 10^−8^ [m·s/kg]
Plant A T1	1.41 (±0.15)	0.11	4.11 (±2.03)
Plant A T2	1.30 (±0.21)	0.16	4.79 (±2.16)
Plant B T1	1.23 (±0.37)	0.30	4.21 (±1.86)

## Data Availability

The data supporting the findings of this study are not publicly available due to security concerns related to the dataset.

## Supplementary Materials

The following supporting information can be downloaded at: https://www.mdpi.com/article/10.3390/membranes14030058/s1, Figure S1: Time Series Variation of K_1_ in Limiting Condition; Figure S2: Time Series Variation of K_2_ in Limiting Condition; Figure S3: Time Series Variation of K_3_ in Limiting Condition; Figure S4: Comparison of Normalized Kernel Density Estimates for K_1_; Figure S5: Comparison of Normalized Kernel Density Estimates for K_2_; Figure S6: Comparison of Normalized Kernel Density Estimates for K_3_; Figure S7: Extracted Cycles in Limiting Condition for Plant A Train 1; Figure S8: Extracted Cycles in Limiting Condition for Plant A Train 2; Figure S9: Extracted Cycles in Limiting Condition for Plant B Train 1; Table S1: Summary of Statistics of K_Lim_ values in different scenarios. Reference [47] is cited in Supplementary Materials.
