# Supplementary material for: Optimizing Air Scouring Energy for Sustainable Membrane Bioreactor Operation by Characterizing the Combination of Factors Leading to Threshold Limiting Conditions
Source: Membranes (Basel). 2024 Feb 23;14(3):58. doi: 10.3390/membranes14030058 (PMC10972311; doi:10.3390/membranes14030058)
Supplement: Supplementary file 1 [file membranes-14-00058-s001.zip › membranes-2816854-supplementary.pdf]

membranes

MDPI

Supplementary Materials

Optimizing Air Scouring Energy for Sustainable Membrane Bioreactor Operation by Characterizing the Combination of Factors Leading to Threshold Limiting Conditions

Changyoon Jun [1,*], Kimia Aghasadeghi [2] and Glen T. Daigger [1]

[1] Department of Civil and Environmental Engineering, University of Michigan, Ann Arbor, MI 48109, USA; gdaigger@umich.edu
[2] Fibracast Ltd., Hannon, ON L0R 1P0, Canada; kimia.aghasadeghi@fibracast.com
* Correspondence: cyjun@umich.edu

Different Version of K Value Equations

The initial idea regarding the relationship between velocity gradient and particle deposition tendency is described by Equation (S1). We have found that the total resistance term is essential to account for broader operational conditions and that the exponents for each side significantly affect the ability to distinguish limiting conditions from sub-limiting conditions, which is the key objective of this metric.

$$\kappa_1\left(\frac{Q_A}{V\ \times\ \mu_{app}}\right)^{0.5} \geq \kappa_2\frac{MLSS\ \times\ Q_P}{A} \tag{S1}$$

There were various experiments conducted to determine a better relationship by changing parameters and exponents on Equation (S1). These attempts are generally summarized by three distinct cases. First, rearranging Equation (S1) by K_{Lim}:

$$K_{1,Lim}\ \leq\ \frac{Q_A}{Q_P}\times\frac{a_s\ \times\ A}{MLSS^2\times\ \mu_{app}\ \times\ Q_P}\ =\ K_1 \tag{S2}$$

The computed value of the right-hand side of Equation (S2) is referred to as K_1 (κ_2^2/κ_1), [s^2/(kg·m)].

Second, when the total resistance term (R_T = (TMP·A)/(μ_w·Q_P)) is added to the right-hand side of Equation (S1) and rearranged by K_{Lim}, which gives:

$$K_{2,Lim}\ \leq\ \frac{Q_A}{Q_P}\times\frac{Q_P\ \times\ a_s\ \times\ \mu_w^2}{MLSS^2\times\ \mu_{app}\ \times\ A\ \times\ TMP^2} = \ K_2 \tag{S3}$$

where K_2 (= $(\kappa_2/\kappa_1)^2$), [(m·s^2)/kg].

Lastly, when the R_T term is added to the right-hand side, balances the exponents on both sides, and eliminates the squared root terms, resulting in Equation (S4):

$$\kappa_1\times\frac{Q_A}{V\ \times\ \mu_{app}} \geq \kappa_2\times\ MLSS\ \times\frac{Q_P}{A}\ \times\ R_T \tag{S4}$$

Equation S4 is then rearranged by K_{Lim}:

$$K_{3,Lim}\ \leq\ \frac{Q_A}{Q_P}\times\frac{a_s}{\mu_{app}\ \times\ MLSS}\times\frac{Q_P\times\ \mu_w}{TMP\ \times\ A} = \ K_3 \tag{S5}$$

where $K_{3,Lim} = \kappa_2/\kappa_1$, [(m·s)/kg], which is corresponding one to Equation (6) in main text.

Comparison Results from Different K Value Equations

Based on the differing behavior observed in Equations (S2), (S3), and (S5), the following two conditions were visually inspected:

K_{Lim} represents a well-defined limiting condition and remains reasonably constant (i.e., its lower boundary is distinct from that of sub-limiting condition).

The general distribution of K_{SubLim} is reasonably dispersed, with less skewness and a wider range of values, to allow for fine adjustments of the air flow according to the actual required air energy.

Figure S1 shows the variation of K values computed by Equation (S2) (i.e., K_1). Both trains of Plant A show a clear differentiation between K_{Lim} and K_{SubLim}, with K_{Lim} located at the lower boundary of K values. However, Plant B shows less distinct separation of limiting conditions, especially during periods when a larger number of limiting conditions is observed. This suggests that an operational condition with frequent fouling cannot be adequately explained if the total resistance term is not taken into consideration.

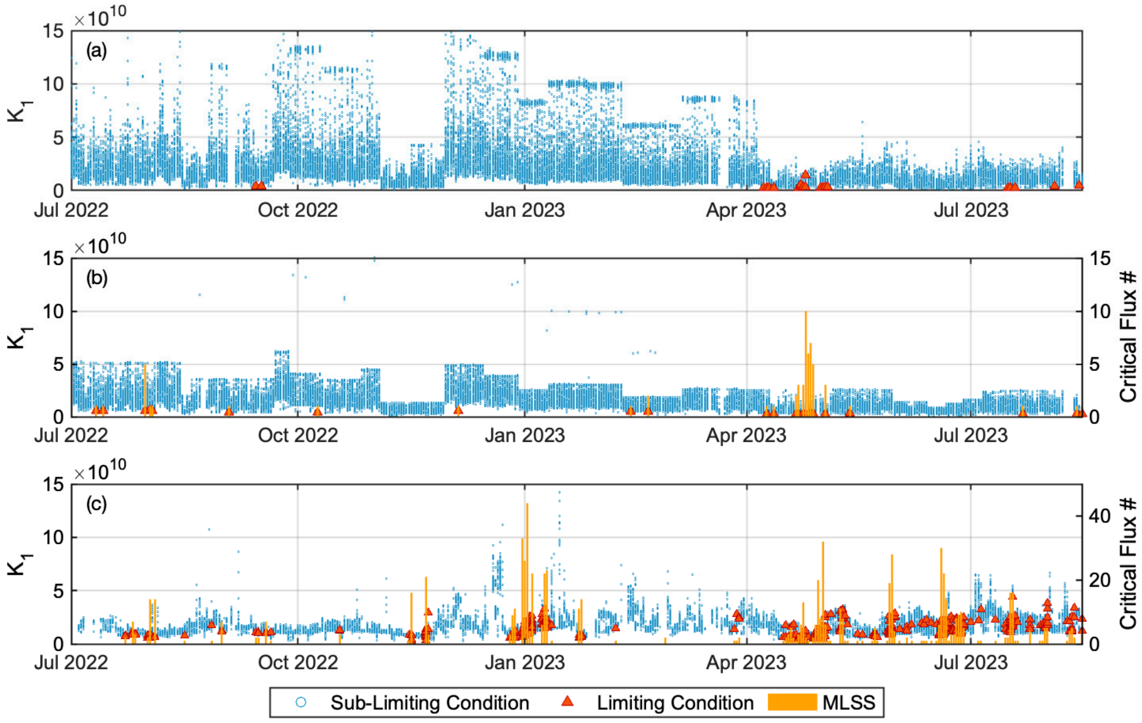

Figure S1. Time Series Variation of K_1 (Equation (S2)) in Limiting Condition (Red Square), Sub-Limiting Condition (Blue Circle), and Number of Observed Limiting Condition (Orange Bar) : (**a**) Plant A Train 1, (**b**) Plant A Train 2, and (**c**) Plant B Train 1.

In case of K_2 (Figure S2), K_{Lim} is generally found at the lower bound of K values in both trains of Plant A and Plant B. Compared to K_1, K_2 has suppressed K values under higher TMP conditions, particularly in Plant B, and the increased vertical variation depends on MLSS (MLSS data is not shown here), suggesting a more sensitive variation to TMP and MLSS.

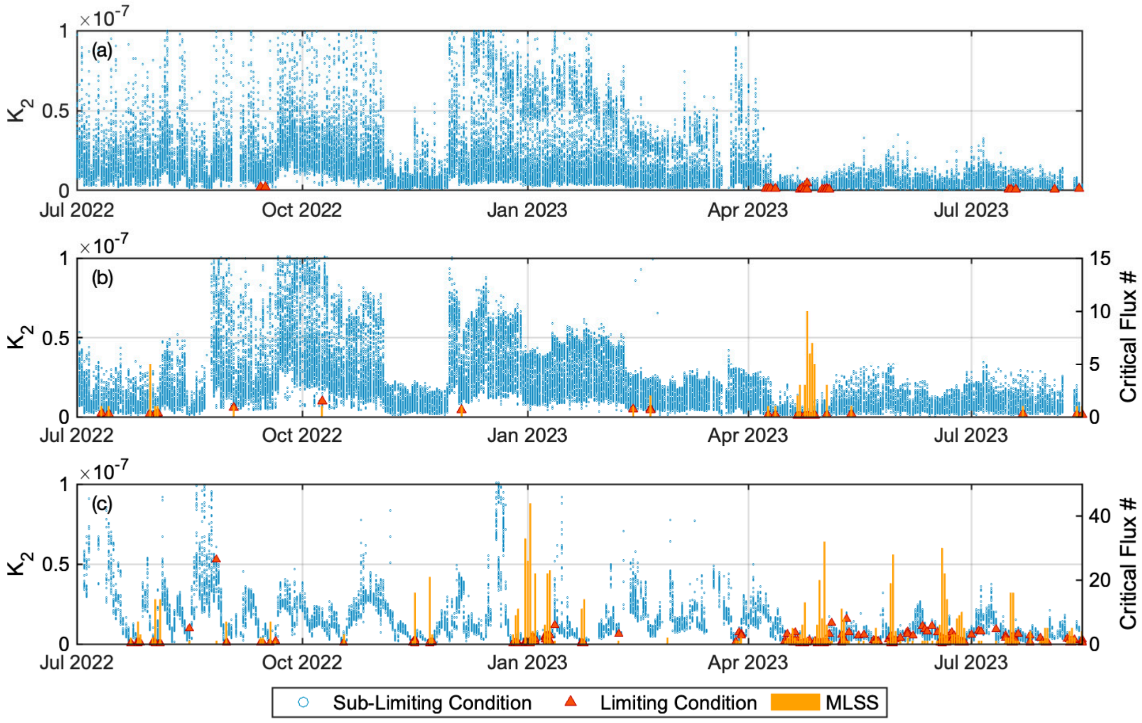

Figure S2. Time Series Variation of K_2 (Equation (S3)) in Limiting Condition (Red Square), Sub-Limiting Condition (Blue Circle), and Number of Observed Limiting Condition (Orange Bar) : (**a**) Plant A Train 1, (**b**) Plant A Train 2, and (**c**) Plant B Train 1.

K_3 has relatively lower sensitivity to MLSS and TMP, showing broader vertical variation of K values. This results in a clearer distinction between limiting conditions and sub-limiting conditions as shown in Figure S3.

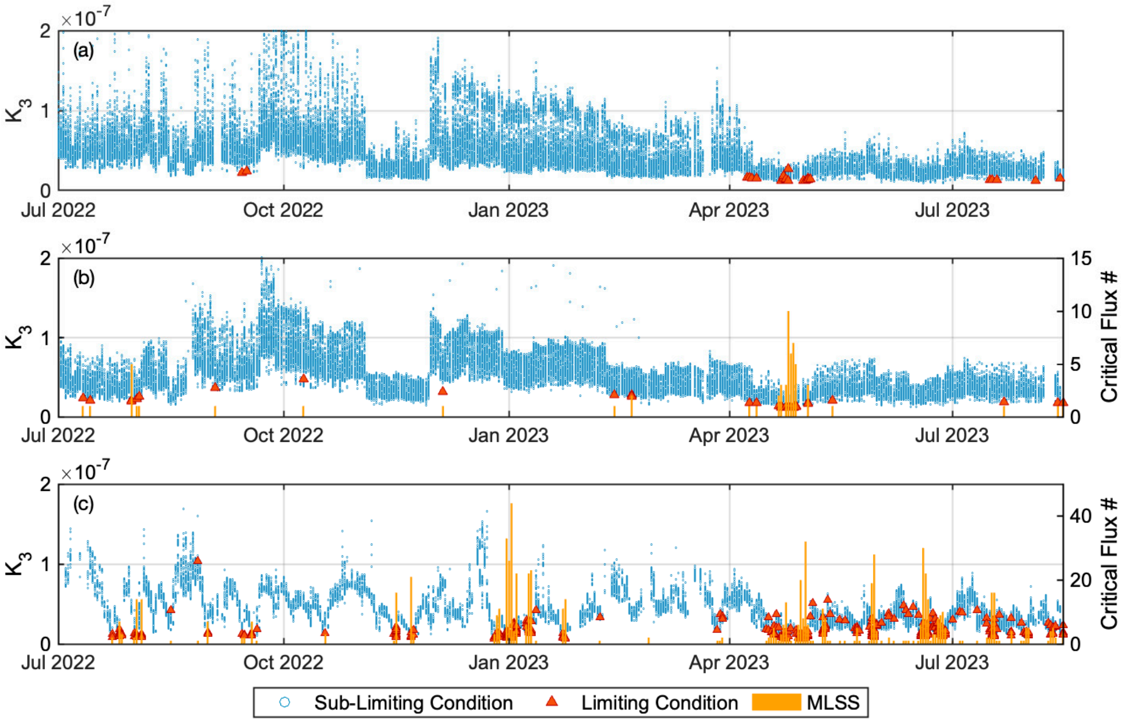

Figure S3. Time Series Variation of K_3 (Equation (S5)) in Limiting Condition (Red Square), Sub-Limiting Condition (Blue Circle), and Number of Observed Limiting Condition (Orange Bar): (**a**) Plant A Train 1, (**b**) Plant A Train 2, and (**c**) Plant B Train 1.

Upon closely inspecting K_{Lim} and K_{SubLim} using Kernel Density Estimation (KDE), it becomes apparent how well K_{Lim} is distinguished between limiting and sub-limiting conditions. Visual inspection of the distributions indicates that K_1 has lower separation ability, especially in Plant B, where the means of the K_{Lim} and K_{SubLim} distributions are closely aligned.

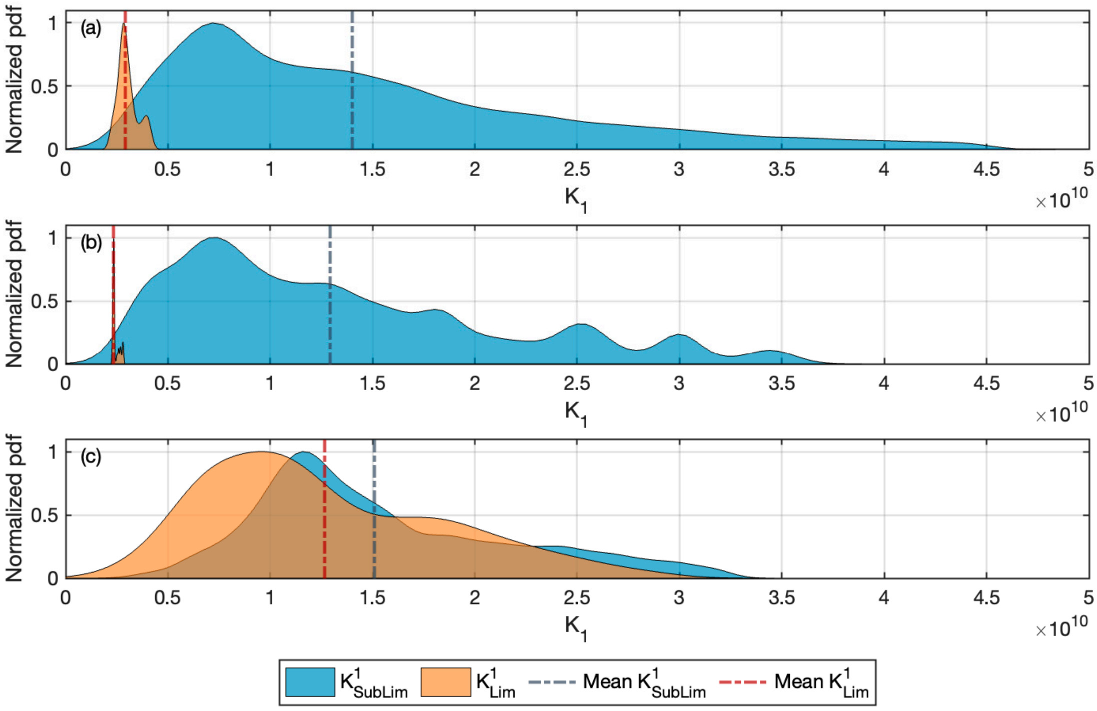

Figure S4. Comparison of Normalized Kernel Density Estimates for K_1 (Equation (S2)) for (**a**) Plant A Train 1, (**b**) Plant A Train 2, and (**c**) Plant B Train 1.

The KDE distribution for K_{SubLim} is a skewed pattern toward to K_{Lim}, and both have a lower boundary near zero (Figure S5), which makes it less efficient to distinguish the K_{Lim} distribution from the K_{SubLim} distribution.

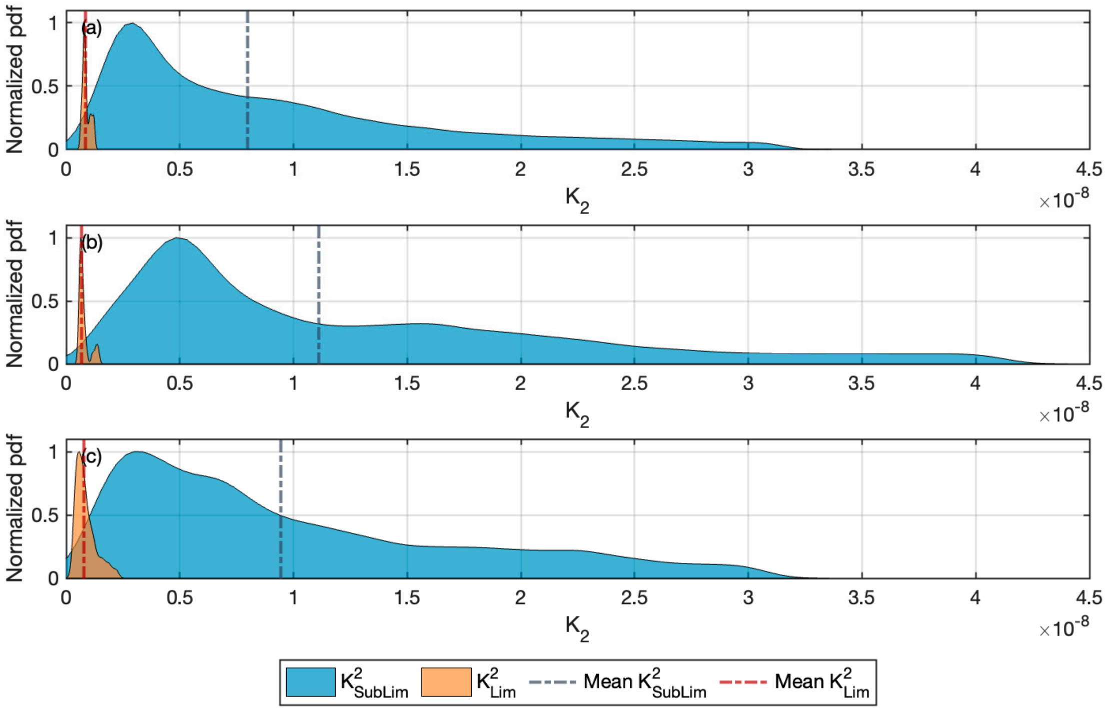

Figure S5. Comparison of Normalized Kernel Density Estimates for K_2 (Equation (S3)) for (**a**) Plant A Train 1, (**b**) Plant A Train 2, and (**c**) Plant B Train 1.

K_3 shows better distinction and clearer differences for both distributions, with less skewness and more reasonable horizontal variation of K_{SubLim} based on mean of K_{SubLim} as a central value.

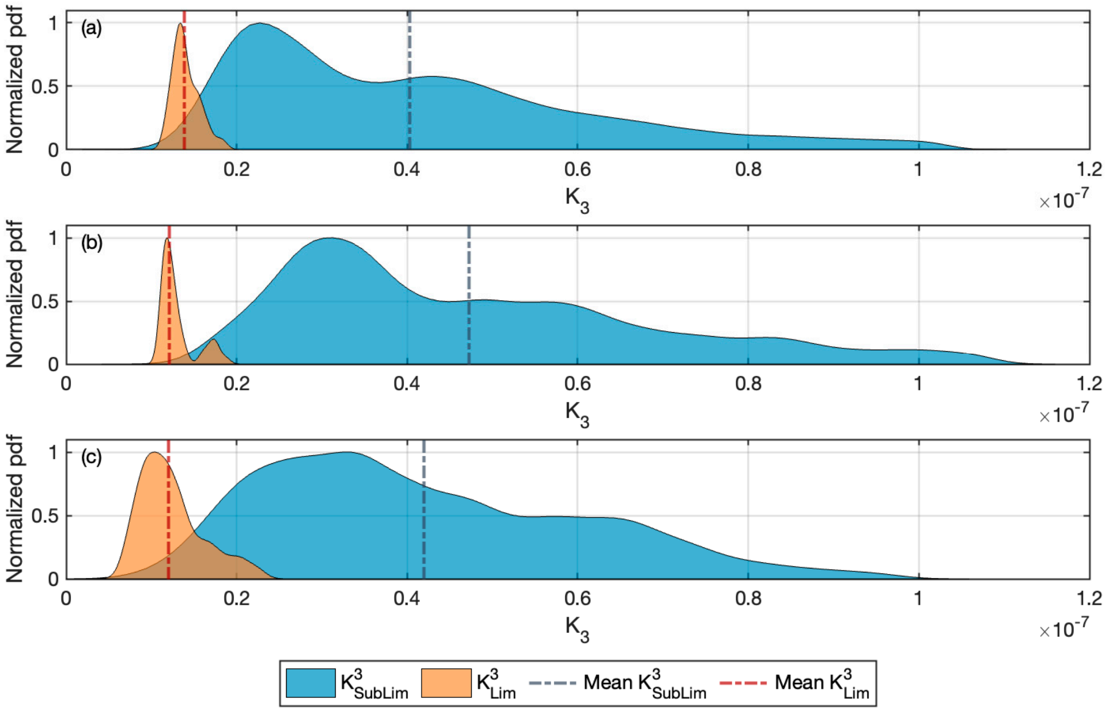

Figure S6. Comparison of Normalized Kernel Density Estimates for K_3 (Equation (S5)) for (**a**) Plant A Train 1, (**b**) Plant A Train 2, and (**c**) Plant B Train 1.

Visual inspection suggests that Equations S2, S3, and S5 produce different results distinguishing the limiting condition and estimating a reasonable variation of K_{SubLim}. Furthermore, it shows that K_3 performs better for these objectives than K_1 and K_2. To ensure a concise estimation of K_{Lim}, Coefficient of Variation (CV) is applied.

Table 1 summarizes the statistics for the determined K_{Lim} values. A lower CV value indicates more concise estimations of $\widehat{K}_{Lim}$ and less variability. Among all other cases, K_3 shows better performance and thus supports the use of this equation for further investigation.

Table S1. Summary of Statistics of K_{Lim} values in different scenarios.

Plant ID	$K_{1,Lim}$		$K_{2,Lim}$		$K_{3,Lim}$	
	**Mean (Std.)**	**CV**	**Mean (Std.)**	**CV**	**Mean (Std.)**	**CV**
Plant A T1	3.0×10^{9} (0.5×10^{9})	0.16	8.9×10^{-10} (1.7×10^{-10})	0.19	1.4×10^{-8} (0.2×10^{-8})	0.11
Plant A T2	2.5×10^{9} (0.2×10^{9})	0.07	7.8×10^{-10} (2.5×10^{-10})	0.31	1.3×10^{-8} (0.2×10^{-8})	0.16
Plant B T1	12.7×10^{9} (5.8×10^{9})	0.45	8.6×10^{-10} (4.4×10^{-10})	0.51	1.2×10^{-8} (0.3×10^{-8})	0.3

Variation of Featured Variables in Limiting Conditions

The featured variables (e.g., TMP, Flux, Permeability) corresponding to the extracted cycles in the detected limiting condition among three different datasets are illustrated in Figures S7 to S9. These figures effectively capture the moments of rapid resistance increase at the given flux, despite some ambiguity (i.e., possible false positive). Permeability [LMH/bar] is computed based on [47]. Note that for Plant B Train 1, due to the large number of cycles in the limiting condition, the extracted cycles are plotted at intervals of 20.

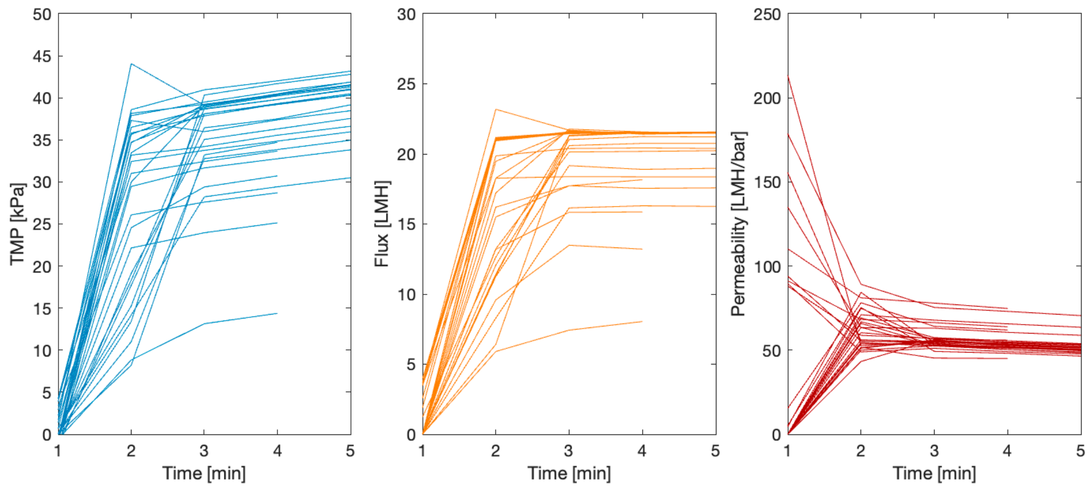

Figure S7. Extracted Cycles in Limiting Condition for Plant A Train 1.

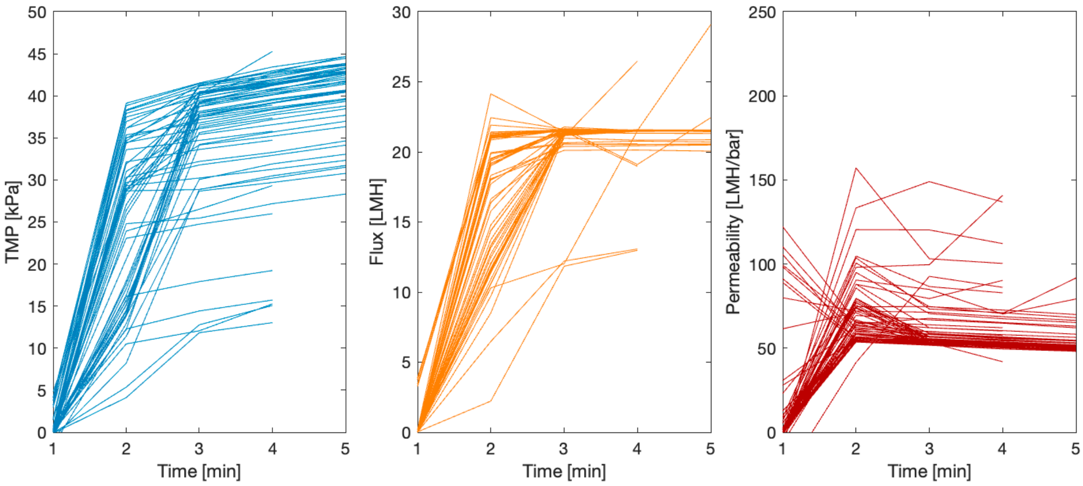

Figure S8. Extracted Cycles in Limiting Condition for Plant A Train 2.

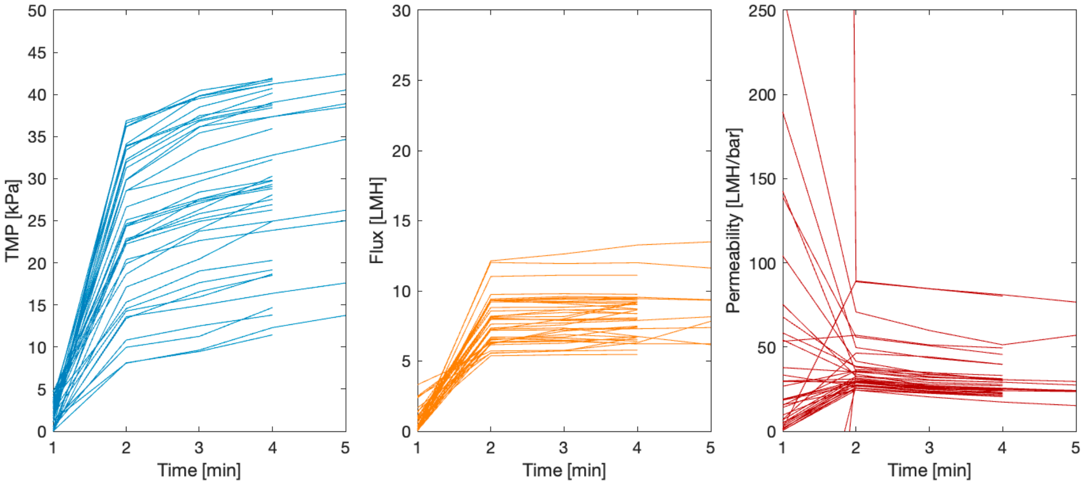

Figure S9. Extracted Cycles in Limiting Condition for Plant B Train 1.

Reference

47. Key MBR operation and maintenance parameters – membrane side. Available online: https://www.thembrsite.com/membrane-processes-basics/membrane-bioreactor-operation-maintenance-flux-tmp-permeability-shear/#:~:text=The%20ratio%20of%20the%20flux,Imperial%20units%20of%20GFD%2Fpsi. (accessed on 31 January 2024).
